# A Deep Dive into Allium Satellite DNAs: Expansion and Characterization of the *Allium cepa* and *Allium fistulosum* Satellitomes

**DOI:** 10.3390/ijms27083476

**Published:** 2026-04-13

**Authors:** Aleksey Ermolaev, Ludmila Khrustaleva, Natalya Kudryavtseva

**Affiliations:** 1 All-Russian Research Institute of Agricultural Biotechnology, 42 Timiryazevskaya Str., 127550 Moscow, Russia; khrustaleva@rgau-msha.ru (L.K.); natalia.kudryavtseva92@gmail.com (N.K.); 2 Center of Molecular Biotechnology, Russian State Agrarian University—Moscow Timiryazev Agricultural Academy, 49 Timiryazevskaya Str., 127550 Moscow, Russia

**Keywords:** satellitome, tandem repeat, satDNA library, *Allium cepa*, *Allium fistulosum*, FISH

## Abstract

Satellite DNA (satDNA) is a family of tandemly repeated non-coding sequences in eukaryotic genomes involved in shaping genome architecture and regulation of various biological functions. Within a species, all satDNA families collectively form the satellitome. Satellitomes of Allium species has been explored only superficially, largely due to enormous genome sizes, high transposable element content, and a general lack of reference genomic resources. The emergence of reference genome assemblies now makes it possible to conduct a more in-depth study. Here, we applied a comprehensive bioinformatics approach to study the satellitomes of *Allium cepa* and *Allium fistulosum*. Using two complementary bioinformatics pipelines along with available reference genome assemblies, we have created the most complete collection of consensus satDNA sequences of *A. cepa* and *A. fistulosum* so far, consisting of 83 and 97 consensus sequences, respectively. The *in silico* analysis of the genomic distribution allowed the identification of 11 novel candidates for cytogenetic marker panels, including chromosome-specific satDNA families. Validation of satDNA using PCR and FISH confirmed the reliability of the created satellitomes. Furthermore, comparative analysis of satDNA genomic organization and abundance provided insights into the evolution of these species satellitomes. These findings provide a foundational resource that will help illuminate the evolutionary dynamics of Allium satellitomes and pave the way for future cytogenetic studies of Allium species.

## 1. Introduction

The genus Allium is characterized by an exceptionally large and variable genome size, ranging from 7.4 to 72.9 Gbp [1]. This genomic expansion is primarily attributable to the substantial amplification of repetitive DNA, which constitutes for over 90% of their genomes [1,2,3,4,5]. This phenomenon is particularly pronounced in the enormous genomes of the bulb onion (*Allium cepa*, ∼16 Gbp/1C) and the bunching onion (*Allium fistulosum*, ∼12 Gbp/1C). Their complex genetic architecture is dominated by Ty3/Gypsy and Ty1/Copia transposable elements [4,6], and an abundance of dispersed repeats [6,7]. The satellitome—the complete collection of tandemly repeated non-coding DNA families within a genome [8]—plays a fundamental role in shaping the genomic landscape. Beyond contributing to genome size, satellite DNA (satDNA) is integral to essential chromosomal structures and functions. In *A. cepa* and *A. fistulosum* it is a key component of centromeres [9,10,11] and telomeres [12] and is involved in higher-order chromosomal organization, such as pericentromeric [13] and subtelomeric regions [4,14,15].

Previous studies of *A. cepa* and *A. fistulosum* satellitomes [4,11,13,16] used a clustering-based approach implemented in the RepeatExplorer2 pipeline [17,18,19]. This pipeline employs graph-based clustering of low-coverage next-generation sequencing reads to identify tandemly organized repetitive sequences [17]. At the time, this was the only available approach for these species because the first chromosome-level genome assemblies for *A. cepa* was published in 2021 [20] and for *A. fistulosum* in 2022 [6], whereas all reported consensus satDNA sequences for these species appeared between 2017 and 2020. Now, despite recent breakthroughs in long-read sequencing and the availability of chromosome-level assemblies for *A. cepa* and *A. fistulosum* [6,20,21,22], their satellitomes remain largely uncharacterized.

The original RepeatExplorer2 pipeline inherently favors the discovery of the most abundant satDNA families, leaving less abundant ones underrepresented [23]. SatDNAs identified using the original RepeatExplorer2 include families associated with key elements of chromosomal landmarks: subtelomere (AceSat01-377 [4], AcepSAT356 [16]), pericentromere (CAT36 [13]), and centromere (AcCen1K [11], TR2CL137 [13]), as well as interstitial families (AceSat02-750 [4], AcepSat750 [16], AcepSAT2500 [16], HAT58 [13]). The only exception is the long centromeric satDNA of *A. fistulosum*, AfCen1K, which was identified via long-range PCR [11] using primers designed for a previously reported centromere-specific sequence Afi11 [10]. Its homologue in *A. cepa*, AcCen1K, was subsequently found using RepeatExplorer2 [11]. Approaches that allow identification of medium- and low-abundance satDNA families, such as RepeatExplorer2 modification SatMiner [8] or assembly-based methods, have never been applied for *A. cepa* and *A. fistulosum*. Consequently, the current satellitomes of these two species remain incomplete, enriched primarily for the most abundant satDNA families and leaving their more low-copy repetitive sequences largely unexplored.

To bridge this gap, we performed genome-wide *de novo* satDNA identification in *A. cepa* and *A. fistulosum* using their available chromosome-level assemblies. Using an ensemble of two complementary bioinformatics pipelines, we identify satDNAs in the genome assemblies of *A. cepa* and *A. fistulosum* and developed comprehensive satellitomes of these species. After extracting candidate satDNA sequences, we performed extensive quality checking and constructed an extended catalogue of 83 and 97 consensus satDNA sequences for *A. cepa* and *A. fistulosum*, respectively. We employed short Illumina reads and long PacBio HiFi reads to estimate and adjust the genomic abundance of identified satDNAs and to determine their genomic organization. Our analysis revealed 128 novel species-specific satDNA families: 66 in *A. cepa* and 62 in *A. fistulosum*. Additionally, we identified 16 satDNA families shared between the two species, 11 of which were previously unreported. Analysis of genomic organization for identified satDNAs revealed novel, highly clustered satDNA families, three in *A. cepa* and eight in *A. fistulosum*. Assembly-based identification of satDNA allowed us to assess the location and distribution of satellites across the genomes and uncovered multiple candidates for novel chromosome-specific satDNA families, which was confirmed by PCR and FISH. In summary, this genome-wide analysis not only substantially expanded the satDNA repertoires of both Allium species but also uncovered multiple candidate satDNAs for chromosome-specific markers, providing valuable tools for future cytogenetic and genomic studies.

## 2. Results

### 2.1. Genome-Wide Identification and Comparison of A. cepa and A. fistulosum Satellitomes

**Figure 1:**
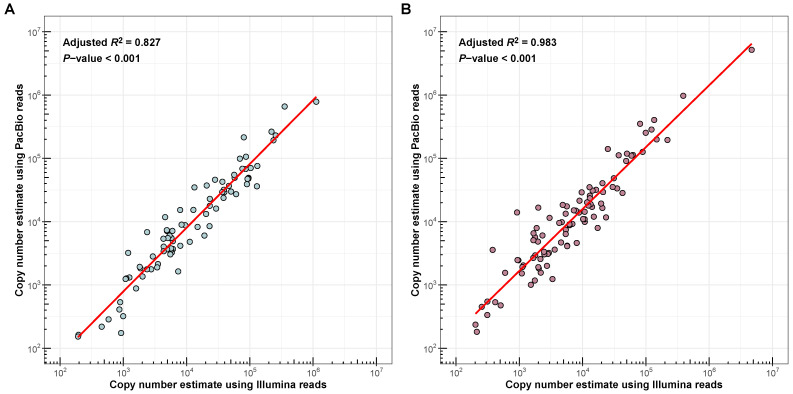
Comparison of *A. cepa* (**A**) and *A. fistulosum* (**B**) satDNA families’ copy number estimation using long HiFi PacBio and short Illumina reads. Each dot represents the satDNA family. The red line represents the regression line.

We performed *de novo* satDNA identification using available high-quality genome assemblies of *Allium cepa* and *Allium fistulosum*. To build comprehensive satellitomes, we processed two genome assemblies each for *A. cepa* [20,22] and *A. fistulosum* [6,21] using two bioinformatics pipelines: TRASH2 and TideCluster. After filtration and deduplication, this approach enabled the compilation of primary satDNAs catalogue consisting of 275 monomer consensus sequences for *A. cepa* and 316 for *A. fistulosum*. Filtering out protein-coding sequences, including those from paralogous genes and transposable elements, left 190 satDNA monomer consensuses for *A. cepa* and 210 for *A. fistulosum*. Removing monomers originating from non-nuclear and rDNA sequences led to the exclusion of one rDNA sequence from satellitomes of each species. The filtered satellitomes were then merged with known satDNA sequences from GenBank (see Section 4). These satDNA consensuses were assembled from short Illumina reads using a clustering approach in the RepeatExplorer2 pipeline [17,18,19] and represent the most abundant and clustered families in the genomes of *A. cepa* and *A. fistulosum*. Following this, final manual deduplication and quality control based on coverage profiles enabled the creation of satDNA collections, comprising 87 consensus sequences for *A. cepa* and 101 for *A. fistulosum*. Subsequently, copy number cross-validation allowed us to identify and exclude four satDNA families in both species due to significant discrepancies between PacBio- and Illumina-based copy number estimates. The final validated satellitomes thus contained 83 satDNA consensus sequences for *A. cepa* and 97 for *A. fistulosum*. Copy number estimates for satDNA families in the final satellitomes, derived from Illumina and PacBio HiFi reads, showed a high squared Pearson correlation coefficient ($R^{2}$) for both *A. cepa* (0.827, *p*-value < 0.001; Figure 1A) and *A. fistulosum* (0.983, *p*-value < 0.001; Figure 1B). Summary information about each satDNA sequence in the satelllitomes and descriptive statistics are presented in Tables S1–S4. FASTA files containing satDNA consensus sequences are openly available in Zenodo at https://doi.org/10.5281/zenodo.18923473.

**Figure 2:**
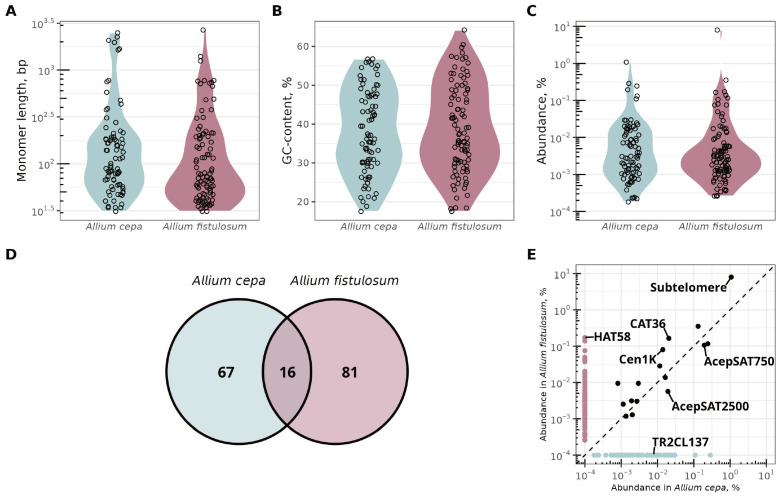
Comparative analysis of of *A. cepa* and *A. fistulosum* satellitomes. The top row shows distributions of monomer length ($log_{10}$ scale; (**A**)), GC-content (linear scale; (**B**)), and genomic abundance estimated from read frequency ($log_{10}$ scale; (**C**)). Each dot represents satDNA family. Below, a Venn diagram details species-specific and shared satDNA consensus sequences (**D**), alongside a scatter plot comparing the genomic abundance ($log_{10}$ scale) of shared and species-specific satDNA sequences between species (**E**). Abundance of species-specific sequences was expressed as a $10^{-4}$ for species where this sequence is absent in order to avoid infinite values in $log_{10}$ scale. Shared satDNA families between species are shown with black dots. Known satDNA families are labeled with their original names. Dots without names represent novel shared satDNA families. The correspondence between the original satDNA names and the names used in this study can be found in Tables S1 and S2.

We identified homologous satDNA sequences across the satellitomes and compared the distribution of monomer length, GC-content, and genomic abundance (Figure 2A–E). The total genomic abundance of satDNA in the constructed collections accounted for 2.84% of the *A. cepa* genome and 8.99% of the *A. fistulosum* genome. Notably, *A. cepa* and *A. fistulosum* share only 16 satDNA families, while the majority are species-specific (Figure 2D, Table S5). The sequence identity for all shared satDNA exceeds 80%. Among the 16 shared repeats, 11 had not been previously identified (Table S5). We found 66 novel species-specific satDNA families in *A. cepa* and 62 in *A. fistulosum*. All previously reported satDNA families in both species—five from *A. cepa* [4,11,16] and three in *A. fistulosum* [11,13]—exhibited non-zero genomic abundance. Interestingly, AcepSAT2500 (AcSat18-2500), previously described as specific to *A. cepa* [11], was also identified in *A. fistulosum* as AfSat30-2674. Most shared repeats, both known and new, have slightly differing consensus monomer lengths (Table S5). The most significant difference (433 bp) is observed in the known long centromeric repeats AcCen1K (AcSat22-1682-cen) and AfCen1K (AfSat8-1249-cen).

Both *A. cepa* and *A. fistulosum* genomes contain a shared subtelomeric repeat (AcSat1-377-subtel/AfSat1-377-subtel), which is the most abundant satDNA family in both species, representing approximately 1.1% of the *A. cepa* genome (937,813 copies/2C) and 8.0% of the *A. fistulosum* genome (4,933,413 copies/2C) (Figure 2C,E, Tables S1, S2 and S5). The least abundant satDNAs were AcSat83-183 in *A. cepa*, comprising 0.00018% of the genome (316 copies/2C) and AfSat97-31 in *A. fistulosum*, comprising 0.00026% of the genome (1954 copies/2C).

Analysis of monomer length distribution revealed that the *A. fistulosum* genome contains more short monomers (<100 bp) repeats, with 57 such families compared to 40 in *A. cepa* (Figure 2A). Conversely, satDNAs with long monomers (>1000 bp) were more common in *A. cepa*, with six families versus three in *A. fistulosum* (Figure 2A). The satDNA monomers GC-content distribution was generally similar in both species, with a slight enrichment of high-GC satDNA families in *A. fistulosum* (Figure 2B). Mean GC-content was nearly identical: 38% for *A. cepa* and 39% for *A. fistulosum* (Tables S3 and S4).

### 2.2. Analysis of Genomic Organization Shows Highly Clustered New satDNA Families

**Figure 3:**
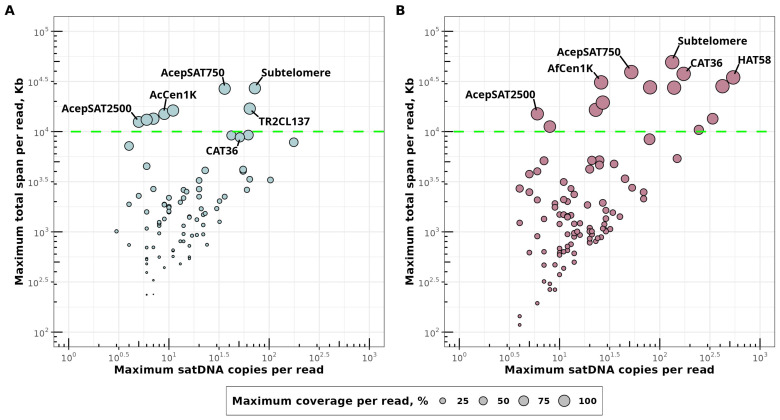
Clustering metrics for satDNA collections of *A. cepa* (**A**) and *A. fistulosum* (**B**). For each plot, the x-axis shows the maximum satDNA monomers copy number per read (MCNPR), and the y-axis shows the maximum total monomers length (span) per read (MTSPR). Both axes are plotted on a $log_{10}$ scale. Each dot represents a satDNA consensus sequence. Dots’ radii represent the maximum coverage of a read by satDNA monomers. The green dashed line represents the 10 kb threshold, above which satDNA families are considered clustered. Known satDNA families are labeled with their original names. Dots without names represent novel satDNA families. The correspondence between the original satDNA names and the names used in this study can be found in Tables S1 and S2.

We evaluated clustering metrics for identified satDNA families in both *A. cepa* and *A. fistulosum* using long HiFi PacBio reads. For each satDNA family in both species, we assessed three metrics: the maximum copy number per read (MCNPR), the maximum total span per read (MTSPR), and the maximum coverage per read (MCOPR) (Tables S6 and S7). Based on these metrics, we detected new highly clustered satDNA families in both species (Figure 3A,B). The satDNA families of *A. fistulosum* are more prone to clustering than those of *A. cepa*, as reflected in the enrichment of satDNA with both high MCNPR and MTSPR (Figure 3B). All previously identified satDNA families in both *A. cepa* and *A. fistulosum* are highly clustered repeats (Figure 3) and have a maximum coverage per read exceeding 90% (Tables S6 and S7).

The subtelomeric repeat (AcSat1-377-subtel/AfSat1-377-subtel) is the most highly clustered satDNA family both in *A. cepa* and *A. fistulosum* (Figure 3A,B, Tables S6 and S7). In *A. cepa*, the clustering metrics of the known pericentromeric repeat CAT36 (AcSat13-243) are significantly lower than those in *A. fistulosum* (AfSat4-221): MCOPR is 64% for AcSat13-243 compared to 99% for AfSat4-221 (Tables S6 and S7). Another satDNA, AcepSAT2500, exhibits similar clustering metrics in *A. cepa* (AcSat16-2500: MCNPR = 5, MTSPR = 12,482 bp, MCOPR = 93%) compared to *A. fistulosum* (AfSat30-2674: MCNPR = 6, MTSPR = 15,030 bp, MCOPR = 80%), but has five times more copies (Tables S1 and S2). SatDNA families with long monomers (>1000 bp) in *A. cepa* (AcSat33-1641, AcSat22-1682-cen, AcSat32-1975, AcSat61-2075, AcSat38-2251, AcSat16-2500) are more prone to clustering than those in *A. fistulosum* (AfSat8-1249-cen, AfSat22-1397, AfSat30-2674), as evidenced by the greater number of families with MCOPR exceeding 90% (Tables S6 and S7).

We identified new clustered satDNA families both in *A. cepa* and *A. fistulosum* satellitomes. The clustering threshold was set at 10 Kb based on MTSPR metrics for known satDNA families (Figure 3, Tables S6 and S7). Analysis revealed several previously unidentified satDNA families above the 10 Kb threshold: three in *A. cepa* (AcSat32-1975, AcSat33-1641, AcSat38-2251) and eight in *A. fistulosum* (AfSat11-741, AfSat13-46, AfSat20-40, AfSat22-1397, AfSat33-370, AfSat44-70, AfSat50-204, AfSat84-725).

### 2.3. In Silico Identification of Chromosome-Specific satDNA Families

**Figure 4:**
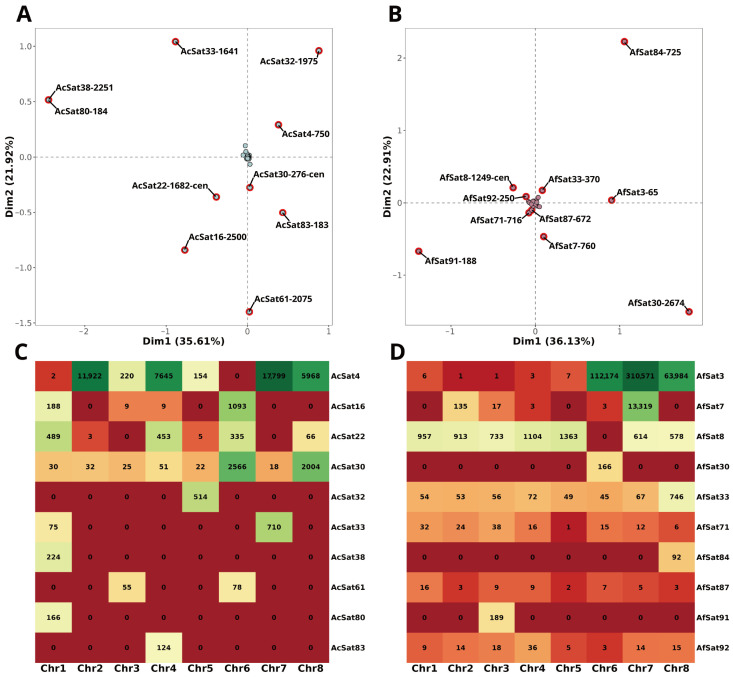
Identification of chromosome-specific satDNA families in *A. cepa* and *A. fistulosum*. The top row shows results of correspondence analysis of satDNA distribution across chromosomes in *A. cepa* (**A**) and *A. fistulosum* (**B**). Intersection of grey dashed lines shows a point of origin (0, 0). The distance from the origin is directly proportional to the irregularity of satDNA distribution across chromosomes. Dots in red borders represent 10 satDNA families with the greatest distance from the origin in each species. The bottom row shows the copy number of these 10 satDNA families across chromosomes in *A. cepa* (**C**) and *A. fistulosum* (**D**).

We evaluated the chromosomal distribution of identified satDNA families in both *A. cepa* and *A. fistulosum* using a bioinformatics approach. This analysis identified several satDNA families with strong chromosome specificity in the reference genome assemblies (Figure 4A,B, Tables S8–S11).

In *A. cepa*, satDNA families exhibiting the most non-uniform distribution across chromosomes include both known and newly characterized repeats (Figure 4A, Table S10). Among these are the well-known centromeric repeats [11], such as AcCen1K (AcSat22-1682-cen), which shows peak copy numbers (PCN) in chromosomes 1, 4, 6, and 8, and TR2CL137 (AcSat30-276-cen), with PCN in chromosomes 6 and 8 (Figure 4C). AcepSAT750 (AcSat4-750) is the most abundant chromosome-specific satDNA in *A. cepa*, with its PCN in chromosomes 1, 4, 7, and 8. Another previously known satDNA, AcepSAT2500 (AcSat16-2500), has its PCN in chromosome 6 and minor PCN in chromosome 1 (Figure 4C). The remaining six satDNA families with the most non-uniform distributions are even more strictly localized. Four of these families (AcSat32-1975, AcSat38-2251, AcSat80-184, AcSat83-183) are strictly confined to a single chromosome, while two families (AcSat33-1641, AcSat61-2075) are found on just two chromosomes (Figure 4C). Notably, all copies of these six satDNA families are located within chromosome scaffolds in the *A. cepa* reference genome (Table S8). Overall, only 33 of the 83 satDNA families (38%) in *A. cepa* are completely assembled into chromosome scaffolds, with no copies remaining in unplaced scaffolds (Table S8).

In contrast, satDNAs of *A. fistulosum* are more uniformly distributed across its chromosomes compared to the more chromosome-specific pattern observed in *A. cepa* (Figure 4B, Table S9). Four previously identified satDNA families displayed a chromosome-specific distribution: HAT58 (AfSat3-65), AcepSAT750 (AfSat7-760), the known centromeric repeat AfCen1K (AfSat8-1249-cen), and AcepSAT2500 (AfSat36-2674). It is noteworthy that AcepSAT2500, previously reported as species-specific for *A. cepa* [11], is identified here in *A. fistulosum*. However, its adjusted copy number in *A. fistulosum* (491 copies/2C) is five times lower than in *A. cepa* (2552 copies/2C) (Tables S1 and S2). Interestingly, its PCN remains in chromosome 6 in both species; however, traces of this satDNA are located in chromosome 1 of *A. cepa* in comparison to chromosome 2 *A. fistulosum* (Figure 4C,D). Another satDNA family, centromeric repeat AfCen1K (AfSat8-1249-cen), was not detected on chromosome 6 of *A. fistulosum* (Figure 4B). Instead, 1024 copies of this satDNA were found in unplaced scaffolds (Table S9). Three satDNA families in *A. fistulosum* (AfSat30-2674, AfSat84-725, AfSat91-188) were found to be strictly chromosome-specific, localized exclusively to chromosomes 6, 8, and 3, respectively (Figure 4D). In the reference *A. fistulosum* genome assembly, only 8 of the 97 satDNA families (8%) are completely anchored in chromosome scaffolds with no copies in unplaced scaffolds (Table S9).

### 2.4. Validation satDNA Families Using PCR and FISH

To validate the identified satDNA, we selected one family from *A. cepa* (AcSat38-2251) and one from *A. fistulosum* (AfSat20-40) based on their distinctive properties. AcSat38-2251 features a very long monomer, strict chromosome-specific localization on chromosome 1 in the reference genome assembly (Table S8), and high clusterization metrics (Table S6). AfSat20-40, a short-monomer satDNA, is present on all chromosomes in the reference genome assembly, but its copy number on chromosome 4 is four-fold higher than on others (Table S9), making this array the most abundant. PCR and FISH validation confirmed the expected product size and the predicted FISH signal positions.

**Figure 5:**
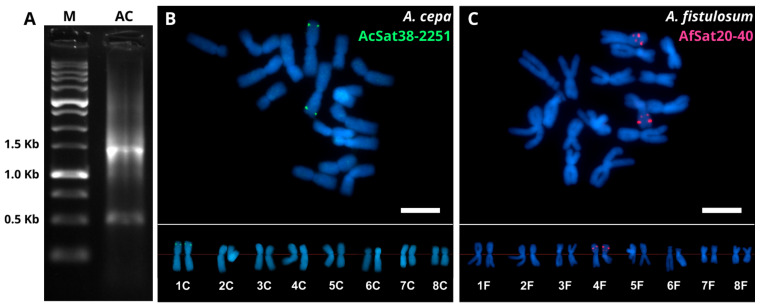
Molecular validation of AcSat38-2251 (**A**,**B**) and AfSat20-40 (**C**) was performed using PCR and FISH. PCR amplification with specific primers for AcSat38-2251 on *A. cepa* DNA yielded an electropherogram displaying a major band at the expected size of ∼1463 bp and a minor band at ∼500 bp (**A**). FISH analysis on mitotic chromosomes revealed bright signals for AcSat38-2251 on the short arm of chromosome 1 (green signals, (**B**)) and for AfSat20-40 on the short arm of chromosome 4 (red signals, (**C**)). Scale bar–10 μm.

In *A. cepa*, PCR using specific primers for AcSat38-2251 yielded a major band of product of the expected size (∼1436 bp) and a minor band of ∼500 bp (Figure 5A). FISH using a probe generated from this PCR product revealed a single locus of twin signals on homogous chromosomes 1 (Figure 5B). The result of FISH mapping is consistent with the reference genome assembly (Table S8).

Validation of AfSat20-40 was performed using FISH with labeled oligonucleotides, since the short length of the monomer did not allow the development of specific primers for PCR amplification. FISH on mitotic chromosomes of *A. fistulosum* detected two loci of twin signals on homologous chromosomes 4 (Figure 5C).

## 3. Discussion

Here we present the most complete catalogues of consensus satDNA sequences for two closely related species: *A. cepa* and *A. fistulosum*. These species have some of the largest genomes among agricultural crops. We performed a comprehensive search of satDNA sequences based on (1) a consensus approach of using all available resources of assembled whole genome sequences of the species; (2) the use of different bioinformatics tools to expand the coverage of repeat searches; (3) careful filtering of all non-satDNA repeats, including gene families, transposable elements and contamination by organelle genomes. This strategy proved to be effective, which was irrefutably confirmed by FISH.

### 3.1. Robustness of the Assembly-Based Satellitomes Construction

The reliable *de novo* identification of satDNA families in two closely related Allium species, *A. cepa* and *A. fistulosum*, required a methodological approach tailored to the challenges of large, repeat-rich plant genomes. To minimize assembly- or tool-specific artifacts, a known limitation of studies relying on a single genome assembly or algorithm [24], we analyzed two high-quality genome assemblies per species using two complementary bioinformatics pipelines, TRASH2 and TideCluster. The resulting satellitomes accurately represent the genomic repeat landscape, as confirmed by the strong correlation between PacBio- and Illumina-based abundance estimates (Figure 1; *A. cepa*: $R^{2}$ = 0.827; *A. fistulosum*: $R^{2}$ = 0.983; *p*-value < 0.001 for both). The notably higher correlation in *A. fistulosum* likely reflects the superior contiguity of its assemblies (see Section 4), which enables more precise reconstruction of tandem repeats within long arrays.

The systematic exclusion of protein-coding sequences, TE-derived sequences, organellar DNA, and rDNA from the initial satDNA candidates pools before constructing the final satellitomes addresses a pervasive contamination problem in large-genome satDNA studies [18,25]. This step is critical, as tandem and dispersed duplications of both genes and pseudogenes have been documented in *A. cepa* and *A. fistulosum* [20,22]. Moreover, in complex genomes, structural similarities between LTR retrotransposon junctions and tandemly organized satellite-like repeats can easily lead to false-positive discoveries, which blurs the number and distribution of satDNAs without rigorous filtering of TE-derived sequences [26]. Furthermore, we removed four families exhibiting substantial discordance between PacBio and Illumina copy number estimates, a signature of putative consensus assembly artifacts. Collectively, these filtering steps establish a benchmark for assembly-based construction of high-confidence satellitomes in species with large genomes, offering a robust framework for future studies.

In our analysis, we used an assembly-based method for identification of satDNA families, but genomic organization and abundance were estimated using unassembled Illumina and PacBio HiFi reads. This approach stems from the main challenge of genome assemblies: accurate placing of satDNA monomers into long arrays [27]. Even newer assemblers still struggle with large repeat-reach genomes, leading to compression of tandem arrays, and their placement into unplaced scaffolds, or their complete exclusion from genome assemblies [27,28]. Using unassembled reads allows assessment of satDNA copy number and genomic organization, mitigating assembly errors.

The comprehensive collection of consensus satDNA sequences of *A. cepa* and *A. fistulosum* established in this study lays a valuable foundation for future studies on the evolution of the Allium genome and opens new avenues for tracking the behavior of individual chromosomes during meiosis in their interspecific hybrids using chromosome-specific satDNAs as cytogenetic markers. The latter is especially compelling given the contrasting distribution of meiotic recombination in these two species [29] and the presence of a large pericentromeric inversion [30], which can cause sterility in interspecific hybrids between *A. cepa* and *A. fistulosum* [31,32].

### 3.2. Differential Amplification and De Novo Emergence Drive Allium Satellitome Divergence

Despite the close phylogenetic relationship between *A. cepa* and *A. fistulosum* [33,34], they share only 16 satellite DNA families, while 67 and 81 families are species-specific, respectively (Figure 2D). This reveals deeply diverged satellitomes between the two species. In both *A. cepa* and *A. fistulosum*, satDNA collectively constitutes only a small fraction of the genome relative to all repetitive elements, which make up approximately 90% and 70% of total genome size, respectively [6,35]. In *A. cepa*, all satDNAs account for 2.84% of the genome size, which is threefold lower than that in *A. fistulosum* (8.99% of the total genome size), despite *A. cepa* having a total genome size 30% larger than that of *A. fistulosum*. This indicates the predominant contribution of transposable elements (TEs) to the genome size of both species.

The satDNA repertoires of these two Allium species present a dual evolutionary picture. On one hand, the 16 shared families, particularly AcepSAT2500, found in *A. fistulosum* as AfSat30-2674 at a five-fold lower copy number, support the library hypothesis [36]. This hypothesis postulates that ancestral low-copy sequences can persist undetected through speciation events and later amplify in independent lineages. On the other hand, the predominance of species-specific families (∼80% in each species) contradicts a strict interpretation of the library hypothesis. Instead, it aligns with the birth-and-death model of satDNA evolution [37,38], where lineage-specific emergence and extinction dominate after speciation. This level of satellitome divergence between *A. cepa* and *A. fistulosum* is similar to that observed in agronomically important barley species *Hordeum chilense* and *H. vulgare* [39]. As in barley, the divergent satDNA profiles in Allium likely originated from differential amplification of shared ancestral satellites coupled with the emergence of new families—sometimes from pre-existing repetitive elements. Remarkably, 11 of the 16 shared staDNA families were novel (Table S5), indicating that previous low-coverage, read-based characterizations of the Allium satellitome only captured the most abundant, highly clustered fraction of the shared satDNA landscape. This underscores the value of assembly-based whole-genome approaches over read-clustering alone for compiling comprehensive satDNA collections in any species.

In our analysis, we observed differences in the length of the consensus sequence for most shared satDNAs, including known families (Table S5). Minor differences may have arisen due to the natural polymorphism of satDNA sequences and the set of representative satDNA sequences used to assemble the consensus. The most significant difference was found for the known centromeric repeats AcCen1K (AcSat22-1682-cen) and AfCen1K (AfSat8-1249-cen): a 433 bp deletion in the *A. fistulosum* variant compared to *A. cepa*. In a previous study, an indel of 632 bp was reported between AcCen1K and AfCen1K [11]. Thus, the observed 433 bp deletion may be a variant of this previously described deletion [11].

### 3.3. Clustering Dynamics and Predictive Power for Cytogenetic Detection

The HiFi PacBio read-based clustering metrics, including the maximum copy number per read (MCNPR), the maximum total span per read (MTSPR), and the maximum coverage per read (MCOPR), proved to be effective proxies for the cytogenetic detectability of satDNA families. In *A. fistulosum*, the general enrichment of highly clustered satDNAs, evident from a greater number of families with high MCOPR and MTSPR values (Figure 3), aligns with its higher total satDNA abundance and may have been driven by recent LTR amplification bursts in this species [6]. All previously characterized satDNA families in both species fall into the highly clustering category (MCOPR > 90%), confirming that earlier studies preferentially captured the most clustered, and therefore most detectable, portion of the satDNA repertoire, while consistently overlooking families with middle or low clustering levels.

A notable discrepancy emerged for the pericentromeric repeat CAT36. While its *A. fistulosum* ortholog (AfSat4-221) achieves near-complete read coverage (MCOPR = 99%), the *A. cepa* counterpart (AcSat13-243) reaches only 64% due to non-satellite DNA interruptions and a lower copy number, resulting in only a weakly dispersed FISH signal on the *A. cepa* chromosomes [13]. However, in *A. cepa* the tandem structure of CAT36 is still detectable as a distinctive ladder on electropherogram [13]. This difference may reflect recently reported centromere instability in *A. cepa* [9], which could lead to disruption of the pericentromeric array.

### 3.4. Chromosome-Specific satDNAs and Limitations of In Silico Localization

The per-chromosome distribution of satellite DNA families revealed striking differences between the two species. In *A. fistulosum*, the majority of satDNA families are distributed more uniformly across chromosomes, whereas in *A. cepa*, multiple families are strictly confined to just one or two chromosomes (Figure 4). Such chromosome-specific satDNA families likely originate from low-copy repeat variants, “seeds”, that undergo a rapid amplification burst, establishing a single chromosomal locus [40,41]. This model is supported by the observation that most novel strictly chromosome-specific satDNA families in both *A. cepa* (AcSat32-1975, AcSat38-2251, AcSat33-1641) and *A. fistulosum* (AfSat30-2674, AfSat84-725) exhibit clustering metrics comparable to known highly clustered satDNAs (Tables S7 and S8).

In both species, these chromosome-specific satDNA families—particularly those confined to a single chromosome—are promising candidates for developing chromosome-specific FISH probes. Such probes allow tracking of individual chromosomes during mitosis and meiosis in *A. cepa*, *A. fistulosum*, and their hybrids. FISH was used for molecular validation of the satDNA chromosome-specific location. We looked at two satDNA families: AcSat38-2251, which was only found on chromosome 1 in the *A. cepa* reference genome assembly (Table S8); and AfSat20-40, which is not specific to one chromosome in the *A. fistulosum* assembly but has four times as many copies on chromosome 4 compared to other chromosomes (Table S9). The chromosome specificity of AcSat38-2251 was fully confirmed at the cytogenetic level (Figure 5B). For AfSat20-40, despite its lack of chromosome specificity at the genomic level, only satDNA arrays on chromosome 4, which harbors the largest copy number, were detected cytogenetically (Figure 5C, Table S9). These results demonstrate that satDNA families lacking chromosome specificity at the genomic level may still function as reliable cytogenetic markers, as their most abundant arrays can be preferentially detected by FISH.

Nevertheless, *in silico* predictions of chromosomal localization warrant caution given the current assembly limitations. Only 38% of satDNA families in *A. cepa* and 8% in *A. fistulosum* are completely located within chromosome scaffolds, while most copies of satDNA families are still found in unplaced scaffolds, showing that it is still difficult to assemble the areas of the genome that have a lot of repeats, especially in pericentromeric and subtelomeric heterochromatin. In the future, using ultra-long Oxford Nanopore reads will help improve Allium genome assemblies, getting us closer to fully understanding how satDNA is spread across chromosomes.

### 3.5. Centromeric satDNA Families

The centromere-associated satDNA families identified in this study, AcCen1K (AcSat22-1682-cen) and TR2CL137 (AcSat30-276-cen) in *A. cepa* and AfCen1K (AfSat8-1249-cen) in *A. fistulosum*, display distinct chromosomal distribution patterns (Figure 5). The long centromere repeat AfCen1K was previously visualized on all *A. fistulosum* chromosomes via FISH [11]. However, our BLASTn-based analysis identified no AfCen1K copies on chromosome 6 in the reference assembly, a discrepancy that may be a technical artifact. A recent study found that the AfCen1K variant on chromosome 6 carries a large deletion relative to the reference variant [9]. Such a deletion could produce fragmented alignments that fall below the query coverage threshold. Alternatively, the absence might reflect an assembly gap. Notably, we detected 1024 copies of AfCen1K in a single unplaced scaffold, an amount comparable to the AfCen1K per-chromosome average of 894 copies (Table S9).

In *A. cepa*, the long centromere repeat AcCen1K (AcSat22-1682-cen) shows PCN on chromosomes 1, 4, 6 and 8 (Table S8). Previously, its location on *A. cepa* chromosomes could not be determined via FISH because of the absence of long arrays in the genome [11]. However, its homologue from *A. fistulosum*, AfCen1K, produced FISH signals on chromosomes 1, 4, 6 and 8 [11]—a pattern that matches the distribution of AcCen1K in the *A. cepa* reference genome assembly determined *in silico*. This result is expected, given the high (>90%) partial sequence similarity between AcCen1K and AfCen1K, except for a large deletion in AfCen1K compared to AcCen1K [11]. Our analysis indicates that AcCen1K exhibits clustering metrics (MTSPR = 14,992 bp; MCOPR = 97.22%, Table S6) sufficient to generate a detectable FISH signal, despite having MTSPR two times lower than that of AfCen1K (30,947 bp; Table S7). However, the copy number of AcCen1K (2746 copies/2C, ∼0.014%) is significantly lower than that of AfCen1K (14,889 copies/2C, ∼0.08%), which may lead to the inability to detect AcCen1K using FISH, despite its high clustering metrics, especially in a centromere, where chromatin accessibility for DNA-probe in FISH is reduced.

Another centromeric repeat, TR2CL137 (AcSat30-276-cen), previously described as chromosome 6-specific based on FISH [11], appears in nearly equal copy numbers on chromosomes 6 and 8 in the reference genome assembly (Table S8). This discrepancy could indicate an assembly artifact. If the assembly is accurate, it raises new questions about the genomic organization of this satDNA family on chromosome 8 and why prior FISH experiments failed to detect it there [11].

These discrepancies between sequence-based and FISH-based detection highlight why no single method can fully capture the complexity of centromere architecture. Based on current understanding of Allium centromere composition, the long-monomer satDNA families identified in this study may represent new centromere-specific variants. This is particularly significant for *A. cepa*, where centromeric repeats have been characterized for only four chromosomes.

## 4. Materials and Methods

### 4.1. Identification satDNA in A. cepa and A. fistulosum

**Figure 6:**
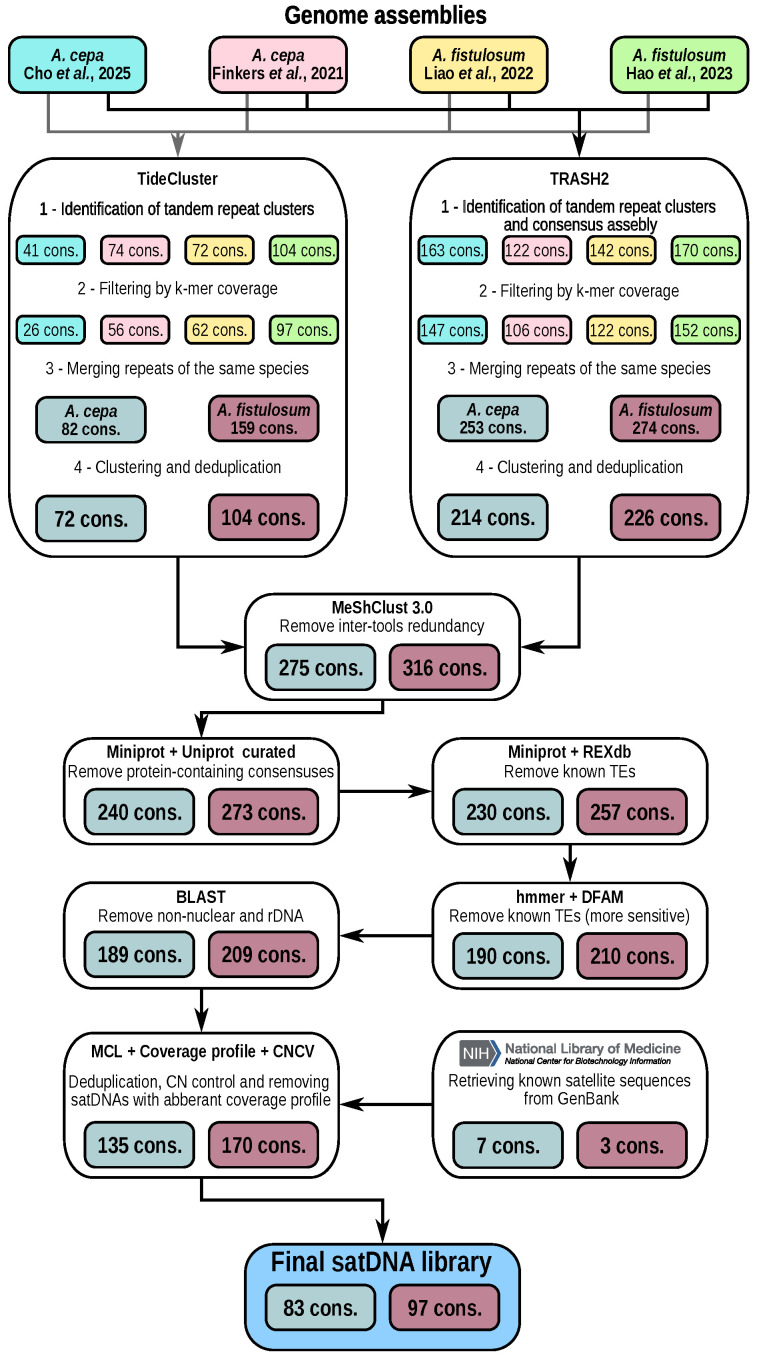
The schematic representation of the pipeline used for *de novo* assembly of *A. cepa* and *A. fistulosum* satellitomes. Results from the different bioinformatics pipelines (TideCluster and TRASH2) and different chromosome-level genome assemblies of *A. cepa* [20,22] and *A. fistulosum* [6,21] were combined to produce a collection containing 83 (*A. cepa*) and 97 (*A. fistulosum*) monomer consensus satDNA sequences. The numbers within the species-specific colored boxes denote the count of consensus sequences retained at each stage of the analysis.

Repetitive DNA was identified *de novo* using two pipelines: TideCluster 1.8.0 (https://github.com/kavonrtep/TideCluster, accessed on 9 September 2025), and TRASH2 (https://github.com/vlothec/TRASH_2, accessed on 9 September 2025). Both of them use genome assembly as input. The analysis pipeline is schematically represented in Figure 6.

All publicly available chromosome-level reference genome assemblies for *A. cepa* [20,22] and *A. fistulosum* [6,21] were obtained from AlliumDB (https://allium.qau.edu.cn, accessed on 9 September 2025) [42]. Assembly statistics were assessed using stats.sh script from the BBTools v39.52-0 (https://bbtools.jgi.doe.gov, accessed on 12 September 2025) and are summarized in Table 1. Before analysis, all assemblies were preprocessed with seqkit 1.4-r122 [43] to ensure sequence uniformity: Soft-masked bases were unmasked, and ambiguous nucleotides were converted to N, standardizing sequence representation for downstream computational analyses.

TideCluster was used on default parameters, except for the “--long” option, which triggers TideCluster to perform three rounds of repetitive DNA detection with increasing monomer size ranges. After each round, identified repetitive DNA is masked in the input sequences for the subsequent round. This approach improves detection of long monomers. For each identified cluster, the best consensus sequence assembled using TAREAN (Tandem Repeat Analyzer) [18] as a part of the TideCluster pipeline was used for further analysis.

TRASH2 was used on default parameters. Since TRASH2 does not assemble monomer consensus sequences, we reconstructed them using TAREAN with default parameters except “--min_kmer_length 7”, as a component of the TideCluster pipeline. To improve assembly quality, identified repeat monomers were reoriented prior to assembly based on k-mer profile using a custom Python 3 script (following the same approach as in TideCluster), thereby standardizing sequence orientation and minimizing artifacts arising from strand ambiguity.

The best assembled consensus sequence was extracted from each cluster identified as both TideCluster and TRASH2 if its k-mer coverage was ≥0.5 and was collected in a single FASTA file. This filtering stage allowed us to select the best-assembled sequences from each cluster and discard clusters in which all sequences were poorly assembled. Collected sequences were deduplicated using MeShClust v3.0 [44] with an 80% identity threshold applied twice: first to remove duplicates from different genome assemblies of the same species, and second to eliminate redundancy between different pipelines.

To eliminate sequences potentially derived from protein-coding regions, the primary sets of satDNA sequences were screened against the UniProt Reviewed (Swiss-Prot) database (Release 2025_04) using miniprot 0.18-r281 [45]. Screening was performed using default parameters, except the maximum intron size was set to $3.6\times \sqrt{reference\;length}$ (-I), and multiple hits for each satDNA consensus were reported if their score exceeded 95% of the best hit score for this sequence (--outs 0.95). The same parameters were then applied to remove transposable element (TE) sequences using the REXdb Viridiplantae v4.0 protein domains database [46].

TE sequences were further filtered using two TE model libraries from the Dfam database (release 3.9, March 2025) [47]. First, we screened the satDNA candidates against the curated Dfam database, which contains manually verified TE families. Second, we screened against uncurated Asparagales-specific TE families extracted from the full Dfam database using FamDB (https://github.com/Dfam-consortium/FamDB, accessed on 28 November 2025) for the taxonomic identifier NCBI:txid73496. Hidden Markov Model (HMM) profiles were built using the HMMER 3.4 [48]. Both screenings were conducted using the dfamscan.pl script (https://www.dfam.org/releases/current/infrastructure/dfamscan.pl.gz, accessed on 17 November 2025) with an E-value threshold of $10^{-3}$, and any sequences matching Dfam profiles under these criteria were removed.

To remove potential contamination from organellar genomes and ribosomal DNA (rDNA), the candidate satDNA sequences were screened against species-specific chloroplast (*A. cepa*—NC_024813.1; *A. fistulosum*—NC_040222.1) and mitochondrial (*A. cepa*—NC_030100.1; *A. fistulosum*—OL347690.1) reference sequences and rDNA using BLAST 2.17.0+ [49]. We retrieved all available rDNA sequences for each species from GenBank using the following queries: for *A. cepa*, “txid4679[Organism:noexp] AND ribosomal[All Fields] NOT chloroplast[All Fields] NOT (mitochondrial[All Fields] OR mitochondrion[All Fields]) NOT shotgun[All Fields]” (73 sequences); for *A. fistulosum*, “txid35875[Organism:noexp] AND ribosomal[All Fields] NOT freshwater[All Fields] NOT chloroplast[All Fields] NOT mitochondrion[All Fields] NOT shotgun[All Fields]” (38 sequences). BLAST searches were performed with an E-value threshold of 0.01, and any sequence with ≥80% alignment coverage to these references was classified as a contaminant and removed.

The final satellitomes for *A. cepa* and *A. fistulosum* were assembled by integrating the filtered consensus sequences with known satDNA sequences from GenBank (Table 2). These families of satellite DNA were predominantly assembled from short Illumina reads using a clustering approach implemented in the RepeatExplorer2 pipeline [17,18,19]. All consensus sequences were manually curated using coverage plots and Markov clustering (MCL). Data for coverage plots were generated for each consensus sequence by mapping short Illumina reads from SRA (SRR12358106 for *A. cepa*, SRR12358105 for *A. fistulosum*) using Bowtie2 2.5.4 [50] with the “--very-sensitive-local” preset. Per-position depth was calculated with SAMtools v1.22.1 [51] and visualized using ggplot2 [52] in R v4.4.1 [53]. For MCL, the satDNA consensuses was self-aligned with BLAST 2.17.0+ [49] (E-value ≤ $10^{-5}$, identity ≥ 80%, query coverage ≥ 80% of the shorter sequence), and bit-scores were used as edge weights for the mcl tool [54] (https://github.com/micans/mcl, accessed on 19 November 2025). Each cluster was then manually curated to eliminate duplicates.

Final quality control involved copy number cross-validation (CNCV) for each satDNA family using both short Illumina reads and long HiFi PacBio reads. We mapped publicly available short-read whole-genome sequencing data (SRA accessions: SRR12358106 for *A. cepa*, SRR12358105 for *A. fistulosum*) to the respective satellitomes using Bowtie2 v2.5.4 [50] with the “--very-sensitive-local” preset. SAMtools v1.22.1 [51] was used to remove unmapped reads (-F 4) and sort the resulting BAM files by reference coordinate. For each satDNA consensus, the number of mapped reads was extracted. Genomic abundance was calculated as the percentage of total reads mapping to that consensus. Absolute copy number per diploid (2C) genome was estimated using the formula:

$$Copy\;number=\frac{2C\;Genome\;size\;(bp)\times Abundance\;(\%)}{Monomer\;length\;(bp)\times 100}$$

where the diploid genome sizes used were 32,830 Mbp for *A. cepa* and 23,275 Mbp for *A. fistulosum* (https://cvalues.science.kew.org/, accessed on 22 January 2026).

We also estimated copy numbers using publicly available HiFi PacBio long reads (SRR24164180 for *A. cepa* and SRR24164236 for *A. fistulosum*). Reads shorter than 10 Kb were excluded. In total, 2,052,627 reads from *A. cepa* and 1,782,980 reads from *A. fistulosum* were used for subsequent analysis. The satDNA consensus sequences were aligned to these reads using BLAST 2.17.0+ [49] with the following thresholds: E-value ≤ $10^{-5}$, identity ≥ 70%, query coverage ≥ 70%, and low-complexity filtering disabled (-dust no). To capture all significant alignments, the maximum number of hits per query was set to 10,000,000. Copy number was calculated as the total number of hits for each consensus.

We used a linear model to assess the relationship between PacBio and Illumina copy number estimates. SatDNA families with significant discrepancies between the two estimates were excluded from further analysis before fitting the model.

The adjusted copy number (ACN) for each satDNA family was calculated as the geometric mean of PacBio and Illumina CNs estimates:

$$ACN=exp(\frac{log(PacBio\;CN)+log(Illumina\;CN)}{2})$$

Genomic abundance was then calculated as the percentage of the genome occupied by all copies (ACN) of each satDNA family.

Finally, the identified satDNA sequences were named according to nomenclature suggested previously [8]: species abbreviation in Repbase (Ac for *A. cepa*, Af for *A. fistulosum*, which is not presented in Repbase, but named in resemblance to *A. cepa*) followed by “Sat”, a numerical index of the satDNA family in the collection in order of decreasing genomic abundance, and the consensus monomer length. A suffix was added to indicate known functional features: “cen” for centromeric repeats and “subtel” for subtelomeric repeats.

### 4.2. Inter-Species Comparative Analysis of Created Satellitomes

Interspecies comparative analysis of consensus sequence homology between the satellitomes of *A. cepa* and *A. fistulosum* was performed using minimap2 v2.28-r1209 [55] with default parameters. Visualization of comparative data was performed using R v4.4.1 [53].

### 4.3. Analysis of the Genomic Organization of the Identified satDNA in A. cepa and A. fistulosum

We analyzed the genomic organization of identified satDNA sequences in *A. cepa* and *A. fistulosum* using publicly available PacBio HiFi long-read data (accessions SRR24164180 for *A. cepa* and SRR24164236 for *A. fistulosum*). Reads shorter than 10 Kb were excluded, resulting in 2,052,627 reads for *A. cepa* and 1,782,980 reads for *A. fistulosum* for subsequent analysis.

SatDNA consensus sequences were aligned against these reads using BLAST 2.17.0+ [49] with the following thresholds: E-value ≤ $10^{-5}$, percent identity ≥ 70%, query coverage ≥ 70%, and low-complexity filtering disabled (-dust no). To ensure all reads with significant alignments were captured, we set the maximum number of alignments per satDNA consensus to 10,000,000.

From the alignment data, we calculated for each satDNA family in each PacBio read the satDNA copy number, total span (the number of bases covered by satDNA monomers), and read coverage. As measures of satDNA clustering in the genome, we used the maximum copy number per read (MCNPR), the maximum total span per read (MTSPR), and the maximum total read coverage per read (MCOPR). Although using long unassembled reads instead of genome assemblies limits resolution due to read length, this approach is sufficient to understand satDNA genomic organization while avoiding the assembly errors common in highly repetitive regions [28].

### 4.4. In Silico Identification of Chromosome-Specific satDNA Families

To identify chromosome-specific satDNA families, we used reference genome assemblies of *A. cepa* [20] and *A. fistulosum* [21]. The copy numbers of satDNA families in the two remaining genome assemblies used in this study [6,22] were also estimated. However, they were not used to identify chromosome-specific satDNA families due to significant discrepancies between observed known satDNA location on chromosome scaffolds and their physical position demonstrated in previous studies [4,11,13,16]. SatDNA consensus sequences from *A. cepa* and *A. fistulosum* were aligned against respective genome assemblies using BLAST 2.17.0+ [49] with the following thresholds: E-value ≤ $10^{-5}$, percent identity ≥ 70%, query coverage ≥ 70%, and with low-complexity filtering disabled (-dust no). The number of BLAST hits per chromosome was counted and visualized using a custom R script. We then identified chromosome-specific satDNA families through correspondence analysis (CA). Hits on unplaced scaffolds were excluded from this analysis. To adjust for multiplicative differences in copy number between chromosomes, we used log-transformed values in the CA. Mahalanobis distances between the origin (coordinates (0, 0)) and the coordinates of each satDNA family were calculated and used as a measure of chromosomal specificity. Larger distances reflect less uniform satDNA copy numbers distribution across chromosomes. CA and visualization were performed using R v4.4.1 [53].

### 4.5. Validation of Identified satDNA Families Using PCR and FISH

For molecular validation using PCR and FISH, we selected one clustered, previously unreported satDNA family from *A. cepa* and one from *A. fistulosum* (Table 3). Plants of *A. cepa* L. var. ‘Myachkovsky 300’ (2*n* = 2*x* = 16) and *A. fistulosum* L. var. ‘Russkiy Zimniy’ (2*n* = 2*x* = 16) were cultivated in the collection of the Center of Molecular Biotechnology at the Moscow Timiryazev Agricultural Academy. Mitotic chromosomes were prepared from root tips following the protocol described by Kudryavtseva et al. [56].

The AcSat38-2251 fragment was amplified by PCR using the primers listed in Table 3. The PCR reaction (25 μL total volume) contained 2.5 μL of 10× Taq Turbo buffer (Evrogen, 25 mM MgCl_2_, pH 8.6), 0.2 mM each dNTP, 0.2 mM each primer, 2.5 U of Taq DNA polymerase (Evrogen, Moscow, Russia), and 100 ng of genomic DNA from *A. cepa* var. ‘Myachkovsky 300’. Amplification was carried out under the following conditions: initial denaturation at 94 °C for 1 min; 30 cycles of 94 °C for 30 s, 58 °C for 15 s, and 72 °C for 2 min; and a final elongation at 72 °C for 5 min. PCR products were analyzed by electrophoresis in a 1% agarose gel in 0.5× TBE buffer at 4 V/cm. The PCR product was labeled with digoxigenin-11-dUTP using a DIG-Nick Translation Mix (Roche, Mannheim, Germany). The chromosomal localization of AcSat38-2251 was determined by FISH as described by Khrustaleva et al. [30].

For AfSat20-40, Cy3-labeled oligonucleotides were synthesized by Evrogen (Moscow, Russia) as listed in Table 3. The small size of the AfSat20-40 monomer did not allow the development of functional PCR primers, so PCR was not performed before FISH. Non-denaturing FISH (ND-FISH) with the developed oligonucleotides was performed following the protocol of Cuadrado and Jouve [57].

Slides were examined under a Zeiss AxioImager M2 microscope (Zeiss, Germany), and images were captured with a Hamamatsu C13440-20CU digital camera (Hamamatsu, Japan). Image processing was performed using Zen 2.6 (blue edition) image analysis software. Karyotype analysis was conducted following the standard nomenclature system proposed by Kalkman [58] and confirmed by the Fourth Eucarpia Allium Symposium [59].

## 5. Conclusions

In this study, we created satellitomes for *A. cepa* and *A. fistulosum* using available genome assemblies and compared their genomic organization between the two species. We identified new clustered satDNA families, including three in *A. cepa* and eight in *A. fistulosum*. Our bioinformatic analysis also revealed candidate chromosome-specific satDNAs and showed that only a fraction of satDNA families are completely represented in chromosome-scale scaffolds, highlighting room for improvement of current reference genome assemblies. The successful FISH visualization of clustered chromosome-specific satDNAs demonstrates that these libraries are a promising source of new cytogenetic markers for both species. These markers can be particularly useful as cytogenetic markers for tracking chromosomes in the meiosis of interspecific hybrids of *A. cepa* and *A. fistulosum*, when chromosome identification using the relative length and centromeric index is difficult. In the future, we will validate the physical chromosomal localization of clustered satDNA families by FISH.

## Figures and Tables

**Table 1:** Summary statistics for each genome assembly used in this study.

Parameter	*A. cepa* [22]	*A. cepa* [20]	*A. fistulosum* [6]	*A. fistulosum* [21]
GC-content, %	33.27	33.75	34.75	35.71
N scaffolds	5347	2099	2875	192
N50 scaffolds, Mb	1400	1056	1386	1405
L50 scaffolds	4	8	4	4

**Table 2:** List of previously published satDNA consensus sequences of *A. cepa* and *A. fistulosum* included in the satellitomes before final manual curation.

Species	Original Name	GenBank ID	Source
*A. cepa*	AceSat02-750	MH017541.1	[4]
*A. cepa*	AceSat01-377	MH017542.1	[4]
*A. cepa*	AcepSAT356	MK770759.1	[16]
*A. cepa*	AcepSAT750	MK770758.1	[16]
*A. cepa*	AcepSAT2500	MK770760.1	[16]
*A. cepa*	AcCen1K	MT374061.1	[11]
*A. cepa*	TR2CL137	MK423913.1	[11]
*A. fistulosum*	AfCen1K	MT374062.1	[11]
*A. fistulosum*	CAT36	KX137122.1	[13]
*A. fistulosum*	HAT58	KX137121.1	[13]

**Table 3:** List of validated satDNA using PCR and FISH.

SatDNA	Probe Type	$5^{\prime }$-$3^{\prime }$ Oligonucleotide/Primer Sequences	Expected PCR Product Size, bp
AcSat38-2251	Labeled PCR product	F: TACCACCAACCCGAATGACCR: TGACGGCTGTGGGATTTGAA	1436
AfSat20-40	Labeled oligonucleotide	CCGGAGTATAAACATCAACTCCGAGTTCCCAGAGCGC	-

## Data Availability

The data presented in this study are openly available in Zenodo at https://doi.org/10.5281/zenodo.18923473 and are included in the article/Supplementary Material. Further inquiries can be directed to the corresponding author.

## Supplementary Materials

The following supporting information can be downloaded at https://www.mdpi.com/article/10.3390/ijms27083476/s1.

## Abbreviations

The following abbreviations are used in this manuscript:

satDNA	Satellite DNA
TE	Transposable Element
LTR	Long Terminal Repeat
MCL	Markov Clustering
SRA	Sequence Read Archive
CNCV	Copy Number Cross-Validation
BAM	Binary Alignment Map
ACN	Adjusted Copy Number
MCNPR	Maximum Copy Number Per Read
MTSPR	Maximum Total Span Per Read
MCOPR	Maximum Total Read Coverage Per Read
CA	Correspondence Analysis
PCN	Peak Copy Numbers
PCR	Polymerase Chain Reaction
FISH	Fluoresent In Situ Hybridization
ND-FISH	Non-denaturing FISH
